# Enhanced antitumor effect of binimetinib in combination with capecitabine for biliary tract cancer patients with mutations in the *RAS/RAF/MEK/ERK* pathway: phase Ib study

**DOI:** 10.1038/s41416-019-0523-5

**Published:** 2019-07-17

**Authors:** Jin Won Kim, Kyung-Hun Lee, Ji-Won Kim, Koung Jin Suh, Ah-Rong Nam, Ju-Hee Bang, Yung-Jue Bang, Do-Youn Oh

**Affiliations:** 1 Department of Internal Medicine, Seoul National University Bundang Hospital, Seoul National University College of Medicine, Seongnam, Republic of Korea; 2 Department of Internal Medicine, Seoul National University Hospital, Seoul National University College of Medicine, Seoul, Republic of Korea; 3 Cancer Research Institute, Seoul National University College of Medicine, Seoul, Republic of Korea

**Keywords:** Biliary tract cancer, Target validation

## Abstract

**Background:**

A phase Ib study of binimetinib and capecitabine for gemcitabine-pretreated biliary tract cancer (BTC) patients was conducted.

**Methods:**

Binimetinib and capecitabine were dosed twice daily on days 1–14, in 3-week cycles. In the dose-escalation (DE) part, three dose levels (DL) were tested (DL1: binimetinib/capecitabine, 15 mg/1000 mg/m^2^; DL2: 30 mg/1000 mg/m^2^; DL3: 30 mg/1250 mg/m^2^).

**Results:**

In the DE part, nine patients were recruited and no dose-limiting toxicity was noted. Therefore, the recommended phase 2 dose was determined as DL3. In the expansion part, 25 patients were enrolled. In total, 34 patients, 25 (73.5%) and 9 patients (26.5%) were second-line and third-line settings, respectively. The 3-month progression-free survival (PFS) rate was 64.0%, and the median PFS and overall survival (OS) were 4.1 and 7.8 months. The objective response rate and disease control rate were 20.6% and 76.5%. In total, 68.4% of stable diseases were durable (> 12 weeks). Furthermore, patients with *RAS/RAF/MEK/ERK* pathway mutations (38.5%) showed significantly better tumour response (*p* = 0.028), PFS (5.4 vs. 3.5 months, *p* = 0.010) and OS (10.8 vs. 5.9 months, *p* = 0.160) than wild type. Most of the adverse events were grade 1/2 and manageable.

**Conclusions:**

A combination of binimetinib and capecitabine shows acceptable tolerability and promising antitumor efficacy for gemcitabine-pretreated BTC, especially in patients with *RAS/RAF/MEK/ERK* pathway mutations.

**Clinical trial registration:**

ClinicalTrials.gov (Identifier: NCT02773459).

## Background

Biliary tract cancer (BTC) arises from bile duct epithelial cells, and is a heterogeneous family of malignant diseases that includes intrahepatic cholangiocarcinoma, extrahepatic cholangiocarcinoma, gallbladder cancer and ampulla of Vater cancer. The incidence of BTC is higher in Korea, China and Thailand than the rest of the world.^1^ Currently, gemcitabine-based chemotherapy is globally considered the first-line treatment for recurrent or metastatic BTC.^2^ However, even with chemotherapy, the median overall survival (OS) of recurrent or metastatic BTC is ~1 year.^2^ There is no solid evidence for the role of second-line chemotherapy, although fluoropyrimidine-based chemotherapy is widely used in practice.^3^ ABC-06 trial has been evaluating the clinical efficacy of second-line mFOLFOX with active symptom control compared with active symptom control alone in BTC. Furthermore, no targeted therapy has been approved for BTC, although many interesting genetic alterations in BTC have been identified.^4^ There is, therefore, a significant unmet need for new and effective BTC treatment strategies.

In BTC, the *RAS/RAF/MEK/ERK* pathway is known to be activated in up to 20–40% of cases, which is mediated by mutations in *KRAS, NRAS, BRAF* and so on.^4,5^ The *RAS/RAF/MEK/ERK* pathway is a series of proteins that mediates communication between the cell surface and nucleus, which has been linked to several vital cellular functions, such as proliferation, apoptosis and metabolism.^6,7^ Therefore, this pathway could have a potential to be a good therapeutic target in BTC. Furthermore, mutations in *RAS* or *BRAF* have been suggested as predictive biomarkers for *MEK* inhibition.^8,9^ In previous clinical trials, selumetinib, an inhibitor of *MEK1/2*, demonstrated interesting activity and acceptable tolerability in patients with metastatic BTC.^10,11^ Similarly, binimetinib is a potent, adenosine triphosphate-uncompetitive, highly selective allosteric inhibitor of *MEK1/2* with on-target activity that has been demonstrated both in vitro and in vivo.^12^ Moreover, phase I study of binimetinib showed a manageable safety profile, target inhibition and dose-proportional exposure.^13^

In this study, we evaluated the preclinical synergistic activity of binimetinib and fluoropyrimidine against BTC cell lines. Supported by the preclinical results, we then conducted a phase Ib study of binimetinib and capecitabine in gemcitabine-pretreated BTC patients to assess the safety and early antitumor activity. Furthermore, we identified genetic alterations to the *RAS/RAF/MEK/ERK* pathway and determined plasma biomarker concentrations.

## Methods

### Preclinical study

The effect of binimetinib, 5-fluorouracil (5-FU) or a combination of these drugs on cell viability was evaluated using eight BTC cell lines. In brief, confluent monolayers, which had been grown in 96-well plates, were exposed to drugs for 72 h. Subsequently, cell viability was measured at 540 nm with a Multiskan GO microplate reader, using a commercially available MTT assay, according to the manufacturer’s directions. The Chou–Talalay method was used to assess a combination effect.^14,15^ Cells were also treated with drugs for 48 h, and the expression of thymidylate synthase (*TS*), programmed death-ligand 1 (*PD-L1*) and β-actin was determined using western blot analysis according to a published protocol.^15^ The experimental material sources are provided in Supplementary Table S1.

### Clinical study design

The clinical study was a phase Ib of dose-escalation and expansion part. The dose-escalation part was conducted as a standard 3 + 3 design to determine the maximum tolerated dose (MTD). The recommended phase 2 dose (RP2D) based on the results of the dose-escalation part was used in the expansion part. The primary endpoint of the dose-escalation part was determination of the MTD, and the secondary endpoints included identification of dose-limiting toxicity (DLT), the RP2D and safety. In the expansion part, the primary endpoint was determination of the 3-month progression-free survival (PFS) rate, and the secondary endpoints were the objective response rate (ORR), response duration, disease control rate (DCR), PFS, OS, safety, quality of life (QOL) and biomarker quantitation.

### Patients

The target population of this study was BTC patients in their second- or third-line treatment setting, who had failed a gemcitabine-based first-line chemotherapy. The major inclusion criteria were age ≥ 20 years; histologically confirmed BTC; unresectable or recurrent disease; prior gemcitabine-based chemotherapy; Eastern Cooperative Oncology Group (ECOG) performance status 0–1; evaluable or measurable lesions by Response Evaluation Criteria in Solid Tumors version 1.1 (RECIST v1.1); adequate bone marrow and organ function; corrected QT interval ≤ 480 ms. The major exclusion criteria were active central nervous system disease; brain metastasis; risk or history of retinal vein occlusion; transplantation history; Gilbert syndrome; major heart disease within 6 months; neuromuscular disease related with elevation of creatine kinase. Biliary drainage was allowed.

### Dosing and dose modification

Binimetinib and capecitabine were orally administered twice daily, on days 1–14, in 3-week cycles. In the dose-escalation part, four predefined dose levels (DLs) were applied (DL-1: binimetinib/capecitabine, 15 mg/800 mg/m^2^; DL1: 15 mg/1000 mg/m^2^; DL2: 30 mg/1000 mg/m^2^; DL3: 30 mg/1250 mg/m^2^). The starting dose was DL1. If no patient experienced DLT in DL3, then DL3 was to be declared as the RP2D. DLT was predefined as grade 4 neutropenia with fever and/or infection; grade 4 neutropenia for ≥ 7 days; grade 3/4 thrombocytopenia with haemorrhage or transfusion; grade 4 thrombocytopenia for ≥ 7 days; grade 3 or 4 non-haematologic adverse events, except alopecia, anorexia, nausea or vomiting; grade 3 or 4 nausea, diarrhoea or vomiting, despite maximum supportive care. Dose reductions of binimetinib to 15 mg twice daily and capecitabine to 75 or 50% of the dose were permitted based on the protocol-defined treatment modifications.

### Assessment of response, adverse events and QOL

Radiologic assessment was completed by computed tomography (CT) every 6 weeks, and tumour response was evaluated based on RECIST v1.1. Routine evaluation, including physical examinations and vital signs, and assessment of adverse events was completed weekly during the first cycle, and then at the end of every cycle. Adverse events were recorded using the NCI—Common Terminology Criteria of Adverse Events version 4.03. QOL was evaluated through the use of the EORTC-QLQ-C30 and EQ5D questionnaires,^16,17^ which were collected at baseline, after cycles 1, 2, 4, 6 and 8, and then after every third cycle until the end of the study.

### Biomarker analysis

All patients were required to provide tumour tissues at screening, and blood samples at screening, after the first cycle, after the second cycle and at disease progression. Genetic alteration was assessed using targeted sequencing by next-generation sequencing (NGS) to determine mutations of the *RAS/RAF/MEK/ERK* pathway. To predict treatment efficacy, interleukin-6 (IL-6) plasma concentrations were evaluated. The plasma concentrations of IL-6 were measured using an enzyme-linked immunosorbent assay (Human IL-6, Quantikine ELISA Kit, R&D Systems, Minneapolis, MN, USA) according to the manufacturer’s instructions. Each sample was analysed in duplicate.

### Statistical analysis

The safety and efficacy analysis were completed in an intent-to-treat population who received at least one dose of binimetinib. Cut-point values of IL-6 at baseline and changes of IL-6 between baseline and the second cycle for OS and PFS predictions were determined by finding the optimal cut point for continuous covariates with time-to-event outcomes.^18^ The results of the EORTC-QLQ-C30 were interpreted in line with the method of Osoba et al.^19^ The PFS was calculated from the date of the first cycle to the development of progressive disease (PD) or death, regardless of the cause. The OS was calculated from the date of the first cycle to death, regardless of the cause. All analyses were performed using PASW Statistics 18 (SPSS Inc., Chicago, IL) and R Statistical Software (R version 3.4.4).

## Results

### Preclinical study

**Fig. 1:**
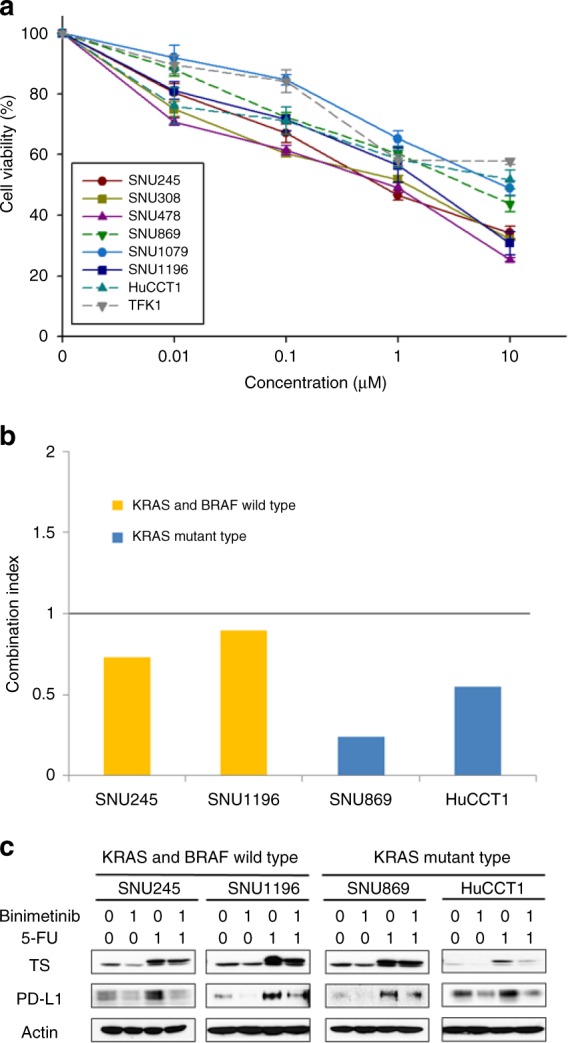
The preclinical efficacy of binimetinib and 5-fluorouracil. **a** Binimetinib demonstrates antitumour activity in biliary tract cancer cell lines. **b** Synergistic properties of the combination of binimetinib and 5-FU are identified in SNU245, SNU1196, SNU869 and HuCCT1 (CI < 1 at Fa = 0.5). **c** *TS*, a 5-FU resistance marker, is upregulated by 5-FU treatment. *TS* levels induced by 5-FU are downregulated by the addition of binimetinib. Similarly, 5-FU-induced expression of *PD-L1* is abrogated by the addition of binimetinib. Actin was included as a loading control. The data are representative of three independent experiments. CI combination index, Fa fraction affected, 5-FU 5-fluorouracil, TS thymidylate synthase, PD-L1 programmed death-ligand 1

Exposure of the BTC cell lines to binimetinib was associated with significant decreases in cell viability (Fig. 1a). The combination of binimetinib and 5-FU in SNU245, SNU1196, SNU869 and HuCCT1 demonstrated synergistic effects (combination index < 1 at the fraction affected = 0.5; Fig. 1b). Of these cell lines, SNU869 and HuCCT1 have the *KRAS* mutation (p.G12D).^20^ To evaluate the underlying synergistic mechanism, protein expression was evaluated by western blot. Accordingly, *TS*, a marker of 5-FU resistance, was upregulated by 5-FU treatment. However, *TS* was downregulated by binimetinib monotherapy (Fig. 1c). When the BTC cells were treated with both binimetinib and 5-FU, the *TS* levels induced by 5-FU were partially downregulated by addition of binimetinib, which may increase the sensitivity of 5-FU. Interestingly, 5-FU induced *PD-L1* expression, and co-administration with binimetinib and 5-FU decreased this expression (Fig. 1c).

### Patients

Nine patients were recruited for the dose-escalation part. None of the patients experienced DLT up to DL3; therefore, the RP2D was determined as DL3 (binimetinib 30 mg, capecitabine 1250 mg/m^2^, twice daily on days 1–14, in 3-week cycles). For the expansion part, 25 patients were enrolled (Supplementary Fig. S1). At the data cut-off time, 22 patients had died, and treatment was ongoing in three patients. The median follow-up duration was 6.8 months (range, 2.0–17.8) and patients received a median of five cycles (range, 1–16).

The median age of enrolled patients was 63 years old (range, 48–73) (Table 1). The primary tumour origins were the gallbladder (*n* = 10, 29.4%), intrahepatic bile duct (*n* = 10, 29.4%), extrahepatic bile duct (*n* = 9, 26.5%) and ampulla of Vater (*n* = 5, 14.7%). Twenty-five (73.5%) patients were in their second-line treatment setting, and nine patients (26.5%) were in their third-line setting. Twelve patients (35.3%) had previously been exposed to fluoropyrimidine as an adjuvant or during a palliative chemotherapy period, and among them, four patients (33.3%) had experienced fluoropyrimidine failure as first- or second-line treatment.

**Table 1:** Patient demographics

Variables	Dose-escalation part (*n* = 9)	Expansion part (*n* = 25)	Total (*n* = 34)
*Age*			
<70	8 (88.9%)	21 (84.0%)	29 (85.3%)
≥70	1 (11.1%)	4 (16.0%)	5 (14.7%)
*Sex*			
Male	3 (33.3%)	17 (68.0%)	20 (58.8%)
Female	6 (66.7%)	8 (32.0%)	14 (41.2%)
*BMI, median (range)*	24.5 (22.1–26.3)	22.2 (17.4–27.7)	23.3 (17.4–27.7)
*ECOG PS*			
0	0 (0.0%)	6 (24.0%)	6 (17.6%)
1	9 (100.0%)	19 (76.0%)	28 (82.4%)
*Tumour origin*			
Gall bladder	4 (44.4%)	6 (24.0%)	10 (29.4%)
Intrahepatic bile duct	2 (22.2%)	8 (32.0%)	10 (29.4%)
Extrahepatic bile duct	2 (22.2%)	7 (28.0%)	9 (26.5%)
Ampulla of Vater	1 (11.1%)	4 (16.0%)	5 (14.7%)
*Disease status*			
Recurrent	3 (33.3%)	11 (44.0%)	14 (41.2%)
Initially metastatic	6 (66.6%)	14 (56.0%)	20 (58.8%)
*Metastatic site*			
Liver	7 (77.8%)	14 (56.0%)	21 (61.8%)
Distant lymph node	2 (22.2%)	12 (48.0%)	14 (41.2%)
Peritoneum	1 (11.1%)	4 (16.0%)	5 (14.7%)
Lung	1 (11.1%)	12 (48.0%)	13 (38.2%)
Bone	0 (0.0%)	3 (12.0%)	3 (8.8%)
Others^a^	0 (0.0%)	2 (8.0%)	2 (5.9%)
*Operation history*	4 (44.4%)	17 (68.0%)	21 (61.8%)
*Chemoradiation history*	2 (22.2%)	5 (20%)	7 (20.6%)
*Adjuvant chemotherapy history*	2 (22.2%)	4 (16.0%)	6 (17.6%)
*Clinical setting*			
Palliative second line	7 (77.8%)	18 (72.0%)	25 (73.5%)
Palliative third line	2 (22.2%)	7 (28.0%)	9 (26.5%)
*Prior fluoropyrimidine exposure*	3 (33.3%)	9 (36.0%)	12 (35.3%)
*Prior fluoropyrimidine failure* ^b^	0 (0.0%)	4 (44.4%)	4 (33.3%)
*CA 19-9, median (range)*	200 (1–73,400)	153 (2–72,100)	157 (1–73,400)

*BMI* body mass index, *ECOG PS* Eastern Cooperative Oncology Group performance status

^a^Adrenal gland, pleural, ^b^of prior fluoropyrimidine exposure

### Adverse events

The majority of the adverse events were both manageable and reversible. Of note, during the first cycle in the dose-escalation part, one G3 neutropenia and one G3 thrombocytopenia occurred, but there was no DLT (Supplementary Table S2). The most common adverse events in the whole population (*n* = 34, dose-escalation and expansion part) were stomatitis (61.7%), oedema (50.0%), nausea (41.2%), papulopustular rash (41.2%), palmar–plantar erythrodysesthesia syndrome (41.2%) and fatigue (41.2%) (Table 2; Supplementary Table S3). There were no ocular adverse events and only one patient experienced grade 4 toxicity (hypokalaemia). There were no treatment-related deaths.

**Table 2:** Common ( ≥ 10%) adverse events (*n* = 34)

Variables, *n* (%)	All grades	Grade 3	Grade 4
Stomatitis	21 (61.7)	1 (2.9)	0 (0.0)
Oedema	17 (50.0)	0 (0.0)	0 (0.0)
Nausea	14 (41.2)	1 (2.9)	0 (0.0)
Papulopustular rash	14 (41.2)	0 (0.0)	0 (0.0)
Palmar–plantar erythrodysesthesia syndrome	14 (41.2)	0 (0.0)	0 (0.0)
Fatigue	14 (41.2)	2 (5.9)	0 (0.0)
Fever	10 (29.4)	1 (2.9)	0 (0.0)
Pruritus	9 (26.5)	0 (0.0)	0 (0.0)
Anorexia	9 (26.5)	0 (0.0)	0 (0.0)
Abdominal pain	8 (23.5)	3 (8.8)	0 (0.0)
Diarrhoea	8 (23.5)	0 (0.0)	0 (0.0)
Vomiting	7 (20.6)	0 (0.0)	0 (0.0)
Dyspnoea	7 (20.6)	1 (2.9)	0 (0.0)
Anaemia	5 (14.7)	4 (11.8)	0 (0.0)
Cholangitis	5 (14.7)	4 (11.8)	0 (0.0)
Upper respiratory infection	5 (14.7)	0 (0.0)	0 (0.0)
Blood bilirubin increased	5 (14.7)	2 (5.9)	0 (0.0)
Back pain	5 (14.7)	0 (0.0)	0 ((0.0)
Neutrophil count decreased	4 (11.8)	1 (2.9)	0 (0.0)
Productive sputum	4 (11.8)	0 (0.0)	0 (0.0)

### Treatment efficacy

**Fig. 2:**
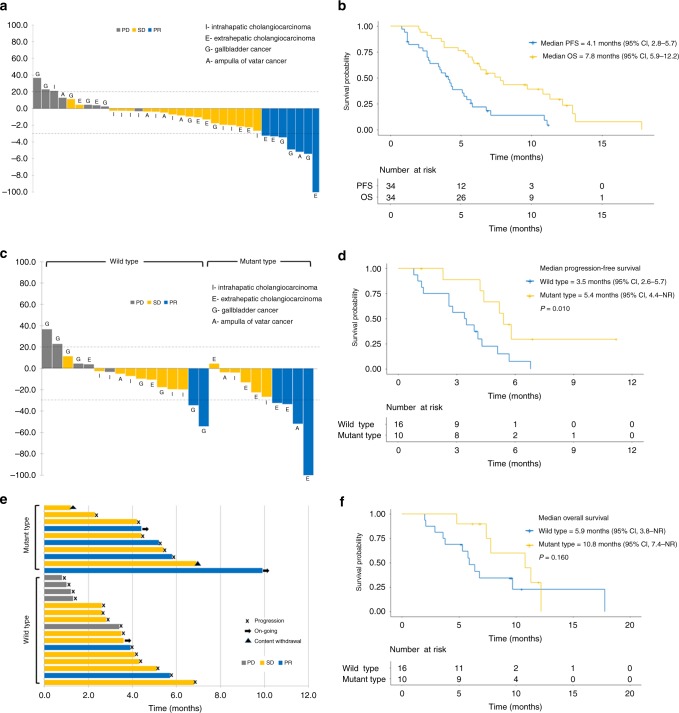
The efficacy of the combination of binimetinib and capecitabine. **a** Waterfall plot of tumour shrinkage, **b** progression-free survival and overall survival are present. Patients with mutant type on *RAS/RAF/MEK/ERK* exhibit better results in **c** waterfall plot, **d** progression-free survival, **e** swimmer plot of treatment duration and **f** overall survival compared with those with wild type. PD progressive disease, SD stable disease, PR partial response, PFS progression-free survival, OS overall survival, CI confidence interval, NR not reached

All patients had at least one measurable lesion. Of 34 patients, 7 patients (20.6%) and 19 patients (55.9%) demonstrated partial response (PR) and stable disease (SD), respectively (Table 3). The ORR and DCR were 20.6% (95% confidence interval (CI), 7.0–34.2) and 76.5% (95% CI, 62.2–90.8), respectively. Twenty-five patients (73.5%) experienced tumour shrinkage with any grade (Fig. 2a), and the median response duration was 4.7 months (95% CI, 2.4–7.0). Of the 19 patients with SD, 13 (68.4%) demonstrated durable disease control with SD duration for >12 weeks. The tumour response was similar between second- and third-line settings (Table 3). Tumour origin also did not alter tumour response. Furthermore, of the four patients who had failed fluoropyrimidine at the first- or second-line setting, one patient (25%) showed PR and the other three patients (75%) showed SD.

**Table 3:** Tumour response, progression-free survival, and overall survival

	Total (*n* = 34)	Second line (*n* = 25)	Third line (*n* = 9)	*P*-value^a^
*Response*				
Complete response	0 (0.0%)	0 (0.0%)	0 (0.0%)	0.988
Partial response	7 (20.6%)	5 (20.0%)	2 (22.2%)	
Stable disease	19 (55.9%)	14 (56.0%)	5 (55.6%)	
Progressive disease	8 (23.5%)	6 (24.0%)	2 (22.2%)	
*Objective response rate*	20.6%	20.0%	22.2%	
*Disease control rate*	76.5%	76.0%	77.8%	
*Progression-free survival*	4.1 months (95% CI, 2.8–5.7)	4.4 months (95% CI, 2.9– 5.9)	3.5 months (95% CI, 2.2–4.8)	0.064
*Overall survival*	7.8 months (95% CI, 5.9–12.2)	7.8 months (95% CI, 5.4–10.2)	6.8 months (95% CI, 5.6–8.0)	0.796

*CI* confidence interval

^a^The comparison between second line and third line

In all patients, the 3-month PFS rate was 64.0% and the median PFS was 4.1 months (95% CI, 2.8–5.7; Fig. 2b). The median OS was 7.8 months (95% CI, 5.9–12.2). Neither the PFS nor OS were significantly different between second- and third-line settings (*p* = 0.064, *p* = 0.796, respectively). There was also no significant difference in the PFS or OS when patients were grouped according to tumour origin (*p* = 0.158, *p* = 0.091, respectively).

### Biomarker analysis

In all patients, tumour tissues were obtained during screening. However, genomic sequence information using NGS techniques was obtained for 26 of the 34 participants (76.5%). Genetic alterations in the *RAS/RAF/MEK/ERK* pathway were identified in 10 (38.5%) of 26 patients (Supplementary Table S4). Seven of these mutations were identified in *KRAS*, one in *NRAS* and two in *MEK*. Furthermore, patients with mutations in the *RAS/RAF/MEK/ERK* pathway responded significantly better to therapy than those with wild type (40.0% vs. 12.5%; Fig. 2c; Supplementary Table S5). Patients with mutant type also showed longer PFS (5.4 vs. 3.5 months; Fig. 2d, e) and OS (10.8 vs. 5.9 months; Fig. 2f) than those with wild type.

**Fig. 3:**
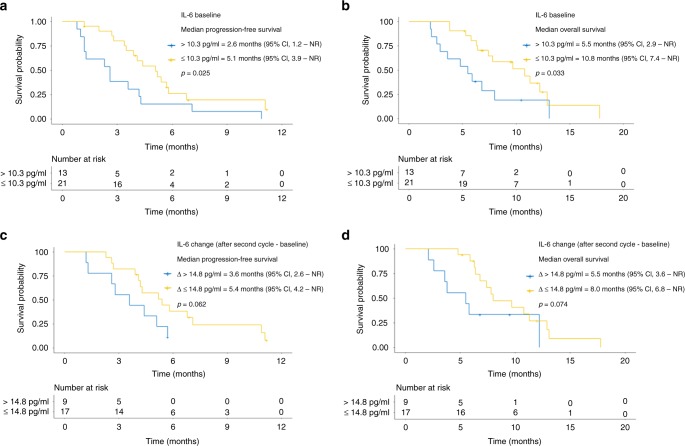
Progression-free survival and overall survival according to IL-6 concentration. **a, b** The baseline plasma concentrations of IL-6 are significantly associated with survival. **c, d** Alterations in the IL-6 concentration from baseline to after the second cycle are associated with survival. IL-6 interleukin-6, CI confidence interval, NR not reached

In terms of IL-6 plasma concentrations, the mean value (± standard deviation) of baseline plasma IL-6 was 11.5 pg/ml (± 12.6). Patients with higher baseline IL-6 showed significantly shorter PFS and OS (*p* = 0.025, *p* = 0.033, respectively; Fig. 3a, b). Similarly, the baseline value of IL-6 was associated with tumour response, that is, IL-6 was higher in PD than PR patients. The mean concentrations were 19.9, 9.2 and 7.7 pg/ml for PD, SD and PR, respectively (*p* = 0.085). With regard to changes between baseline and after the second cycle, a greater increase in the IL-6 concentration (Δ > 14.8 pg/ml) was associated with shorter PFS and OS (Fig. 3c, d). Furthermore, the plasma concentrations of IL-6 when PD was confirmed were also significantly increased relative to baseline (mean ± standard deviation: 32.0 ± 29.8 vs. 9.0 ± 5.9 pg/ml, respectively; paired *t* test, *p* = 0.008).

### QOL

Based on the EORTC-QLQ-C30 questionnaire, most QOLs regarding global health status and functioning were altered between the degrees of ‘a little’ to ‘very much’ as the cycles proceeded (Supplementary Table S6). QOLs related to symptoms demonstrably improved at some time points. Compared with best status to baseline, role functioning and financial difficulties improved with ‘a little’ degree (*p* = 0.028 and *p* = 0.032, respectively), and QOL related to pain improved with ‘moderate’ degree (*p* = 0.039). The EQ5D questionnaire also demonstrated alterations of the QOL throughout the treatment period (Supplementary Table S7).

## Discussion

This study is the first proof-of-concept trial to evaluate the safety and efficacy of binimetinib in combination with capecitabine for gemcitabine-pretreated BTC, and was supported by preclinical data suggestive of the synergism between binimetinib with fluoropyrimidine. This combination demonstrated promising antitumour efficacy, especially in BTC patients with mutations in the *RAS/RAF/MEK/ERK* pathway.

A previous phase Ib study of binimetinib monotherapy in advanced or metastatic BTC patients reported an ORR of 8% and a DCR of 51%, as well as PFS of 2.1 months and OS of 4.8 months, respectively.^21^ Similarly, in a phase II study of selumetinib for metastatic BTC, the ORR, DCR, PFS and OS were 12%, 80%, 3.7 months and 9.8 months, respectively.^10^ In contrast, the ORR, DCR, PFS and OS were 20.6%, 76.5%, 4.1 months and 7.8 months, in this study. Considering that our study consisted of patients in their second- (73.5%) and third-line settings (26.5%), the efficacy of this study may be better than the aforementioned studies, in which more than half of the patient populations were chemotherapy-naive in a metastatic setting (57 and 75%, respectively). Furthermore, a systematic review, of patients in the second- and third-line setting for BTC, described ORR and DCR of <10% and <50%; the PFS and OS were around 3 months and 6 months.^3^ Moreover, our previous study, which included both second- and third-line setting BTC patients treated with a 5-FU-based combination treatment (infusional FAM regimen), had a similar population to this study, and reported PFS and OS values of 2.4 months and 6.1 months, respectively.^22^ Considering these results, the efficacy of binimetinib and capecitabine in this study is very encouraging and is likely attributed to the synergism between binimetinib and capecitabine. Indeed, preclinical experiments demonstrated the downregulation of *TS* and *PD-L1* induced by 5-FU in response to binimetinib. Synergistic properties between a MEK inhibition and fluoropyrimidine have also been reported for other cancer types.^23–25^ Interestingly, in this study, all patients who had previously failed fluoropyrimidine-based chemotherapy achieved either a PR or SD with this combination, which is likely attributed to synergism.

Of note, activation of the MEK pathway, such as through *RAS* or *BRAF* mutations, has been reported as a predictive marker for the success of *MEK* inhibitors.^8,9^ In this study, genetic mutations within the *RAS/RAF/MEK/ERK* pathway were identified in 10 (38.5%) out of 26 patients, whose NGS data were obtained. This incidence was in accordance with previous studies.^4,5^ Importantly, this study demonstrates that a tissue-based biomarker selection strategy for BTC patient management or enrolling BTC patients into clinical trials is doable and feasible. Interestingly, patients with mutations in the *RAS/RAF/MEK/ERK* pathway were associated with a higher ORR and longer survival than those with wild type. Considering that our patients were in the second- or third-line setting of BTC, these results of ORR (40%), PFS (5.4 months) and OS (10.8 months) were very promising in patients with mutant type of *RAS/RAF/MEK/ERK* pathways. Therefore, mutations in the *RAS/RAF/MEK/ERK* pathway could be a predictive biomarker for effective binimetinib treatment of BTC.

Immune modulation is another antitumour mechanism of *MEK* inhibitors,^26^ as *MEK* inhibition was reported to reduce the secretion of IL-6, which is associated with BTC tumour growth.^26–29^ In this study, higher baseline concentrations of IL-6 were associated with worse prognosis, which is similar to the results from other studies regarding the prognostic value of IL-6.^30,31^ Furthermore, after the second cycle of treatment, patients with a larger increase were associated with worse prognosis. Therefore, early comparative determination of IL-6 between baseline and after treatment may predict disease outcomes in the binimetinib treatment.

In the previous phase I study of binimetinib monotherapy, 60 mg twice daily was identified as the MTD.^13,21^ However, due to the frequent treatment-related ocular toxicity at this MTD, 45 mg twice daily was used as the RP2D.^13,32^ In this study, predefined four DLs were tested, and no DLT was observed. The highest DL (binimetinib 30 mg, capecitabine 1250 mg/m^2^, twice daily on days 1–14, in 3-week cycles) was determined as the RP2D. This dosage of binimetinib was relatively low in comparison with the monotherapy study, and we introduced a 1-week drug holiday in 3-week cycles. This combination dosing schedule, which allowed full-dose capecitabine, was tolerable and adverse events were manageable. There was also no ocular toxicity in our study. Furthermore, this combination resulted in promising efficacy outcomes.

In conclusion, the preclinical synergistic activity of binimetinib with 5-FU against BTC was demonstrated to support the clinical development of this combination. We next completed a clinical phase Ib trial, assessing the combination of binimetinib and capecitabine in patients with gemcitabine-pretreated advanced BTC. The RP2D was determined during the dose-escalation part of the trial and was identified as 30 mg binimetinib with 1250 mg/m^2^ capecitabine, twice daily on days 1–14 in 3-week cycles. The drug combination was well tolerated, associated with manageable adverse events, and demonstrated promising antitumour efficacy, especially in patients with *RAS/RAF/MEK/ERK* pathway mutations. These findings support future clinical development of *MEK* inhibition strategies for BTC management.

## Supplementary information

supplementary table and figure

## Data Availability

All data and materials are available by inquiring to the corresponding author.
