# Supplementary material for: Enhanced antitumor effect of binimetinib in combination with capecitabine for biliary tract cancer patients with mutations in the RAS/RAF/MEK/ERK pathway: phase Ib study
Source: Br J Cancer. 2019 Jul 17;121(4):332–9. doi: 10.1038/s41416-019-0523-5 (PMC6738070; doi:10.1038/s41416-019-0523-5)
Supplement: Supplementary file 1 — supplementary table and figure [file 41416_2019_523_MOESM1_ESM.docx]

**Supplementary Figures and Tables**

**Supplementary Table S1. Material sources for preclinical studies**

| **Materials** | **Sources** |
| --- | --- |
| SNU245, SNU308, SNU478, SNU869, SNU1079, and SNU1196 | Korean Cell Line Bank, Seoul, Korea |
| TFK1 and HuCCT1 | RIKEN BioResource Center, Ibaraki, Japan |
| Binimetinib | Novartis, Basel, Switzerland; Selleck Chemicals LLC, Houston, TX, USA |
| 5-fluorouracil | Ildong Pharmaceutical Co., Seoul, Korea |
| 3-(4,5-dimethylthiazol-2yl)-2,5-diphenyltetrazolium bromide solution | Sigma-Aldrich, St. Louis, MO, USA |
| Primary anti-thymidylate synthase antibody for western blot | Cell Signaling Technology, Beverley, MA, USA |
| Primary anti-programmed death-ligand 1 antibody for western blot | Cell Signaling Technology, Beverley, MA, USA |
| Primary anti-β-Actin antibody for western blot | Sigma-Aldrich St. Louis, MO, USA |
| Secondary anti-mouse antibody for western blot | Thermo Fisher Scientific. Waltham, MA, USA |
| Secondary anti-rabbit antibody for western blot | Thermo Fisher Scientific, Waltham, MA, USA |
| Multiskan Go microplate reader | Thermo Fisher Scientific, Waltham, MA, USA |
| CalcuSyn software | Biosoft, Ferguson, MO, USA |

**Supplementary Fig S1. CONSORT diagram**

**
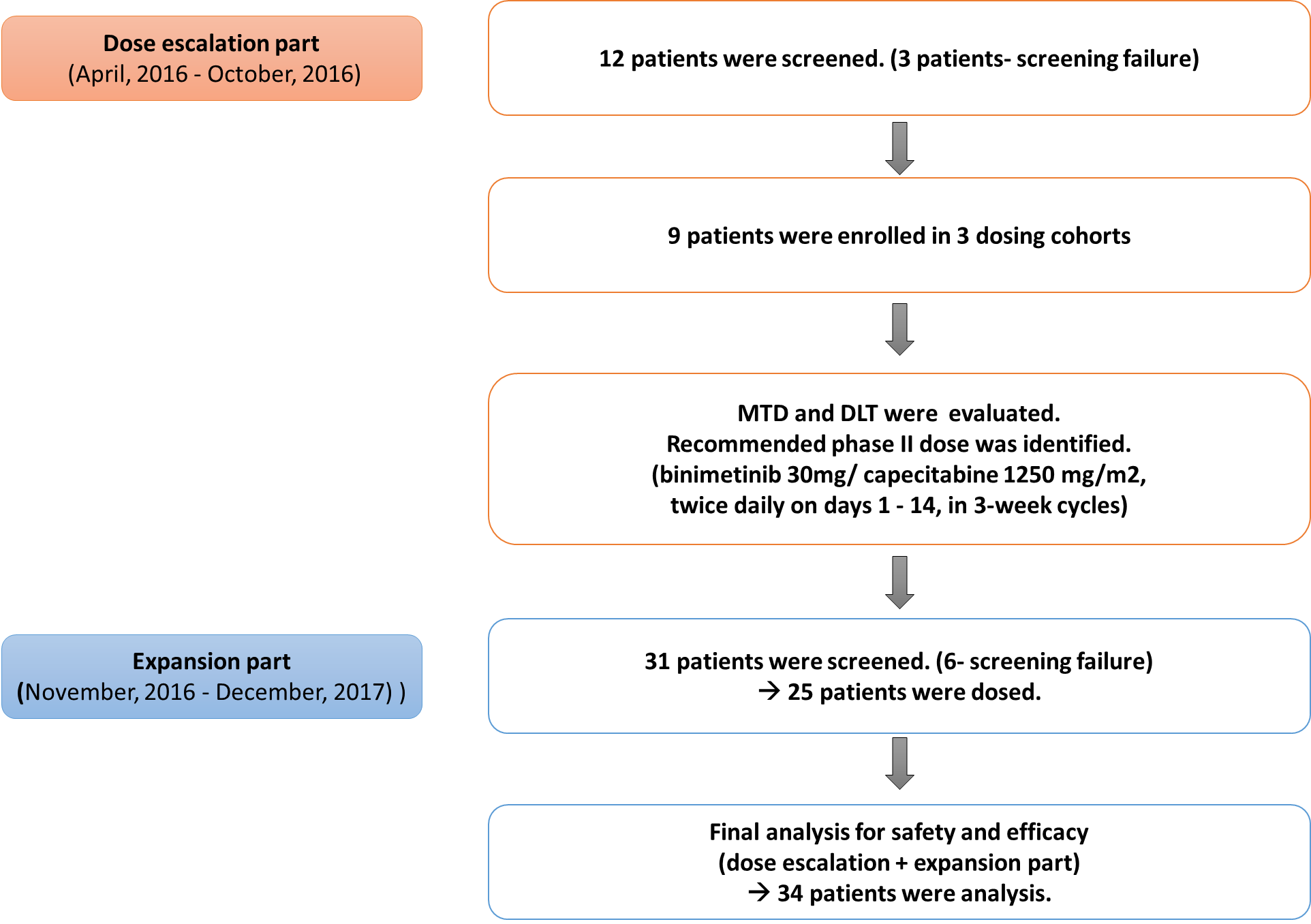
**

**Supplementary Table S2. Adverse events in the dose-escalation part (n = 9)**

| **Variables** | **DLT-evaluable period (first 3 weeks), patients** | | | | **Total** |
| --- | --- | --- | --- | --- | --- |
| **Grade** | **G1** | **G2** | **G3** | **G4** |  |
| Platelet count decreased | 0 | 1 | 1 | 0 | 2 (22.2%) |
| Neutrophil count decreased | 0 | 0 | 1 | 0 | 1 (11.1%) |
| Stomatitis | 5 | 0 | 0 | 0 | 5 (55.6%) |
| Edema | 5 | 0 | 0 | 0 | 5 (55.6%) |
| Back pain | 4 | 0 | 0 | 0 | 4 (44.4%) |
| Anorexia | 3 | 0 | 0 | 0 | 3 (33.3%) |
| Nausea | 2 | 0 | 0 | 0 | 2 (22.2%) |
| Papulopustular rash | 2 | 0 | 0 | 0 | 2 (22.2%) |
| Pruritus | 1 | 0 | 0 | 0 | 1 (11.1%) |
| Fever | 1 | 0 | 0 | 0 | 1 (11.1%) |
| Headache | 1 | 0 | 0 | 0 | 1 (11.1%) |
| Pain in extremity | 1 | 0 | 0 | 0 | 1 (11.1%) |
| Peripheral sensory neuropathy | 1 | 0 | 0 | 0 | 1 (11.1%) |
| Productive sputum | 1 | 0 | 0 | 0 | 1 (11.1%) |
| Productive cough | 1 | 0 | 0 | 0 | 1 (11.1%) |
| Diarrhea | 1 | 0 | 0 | 0 | 1 (11.1%) |
| Flank pain | 1 | 0 | 0 | 0 | 1 (11.1%) |

DLT, dose-limiting toxicity; G, grade

**Supplementary Table S3. All adverse events (n = 34)**

| **Variables** | **G1** | **G2** | **G3** | **G4** | **Total (n)** | **Total (%)** |
| --- | --- | --- | --- | --- | --- | --- |
| **Hematologic adverse events** |  |  |  |  |  |  |
| Anemia | 0 | 1 | 4 | 0 | 5 | 14.7 |
| Neutrophil count decreased | 1 | 2 | 1 | 0 | 4 | 11.8 |
| White blood cell decreased | 0 | 3 | 0 | 0 | 3 | 8.8 |
| Platelet count decreased | 0 | 1 | 1 | 0 | 2 | 5.9 |
| **Non-hematologic adverse events** |  |  |  |  |  |  |
| Stomatitis | 14 | 6 | 1 | 0 | 21 | 61.7 |
| Edema | 14 | 3 | 0 | 0 | 17 | 50.0 |
| Nausea | 11 | 2 | 1 | 0 | 14 | 41.2 |
| Papulopustular rash | 12 | 2 | 0 | 0 | 14 | 41.2 |
| Palmar-plantar erythrodysesthesia syndrome | 11 | 3 | 0 | 0 | 14 | 41.2 |
| Fatigue | 5 | 7 | 2 | 0 | 14 | 41.2 |
| Fever | 7 | 2 | 1 | 0 | 10 | 29.4 |
| Pruritus | 9 | 0 | 0 | 0 | 9 | 26.5 |
| Anorexia | 8 | 1 | 0 | 0 | 9 | 26.5 |
| Abdominal pain | 4 | 1 | 3 | 0 | 8 | 23.5 |
| Diarrhea | 7 | 1 | 0 | 0 | 8 | 23.5 |
| Vomiting | 7 | 0 | 0 | 0 | 7 | 20.6 |
| Dyspnea | 5 | 1 | 1 | 0 | 7 | 20.6 |
| Cholangitis | 0 | 1 | 4 | 0 | 5 | 14.7 |
| Upper respiratory infection | 1 | 4 | 0 | 0 | 5 | 14.7 |
| Blood bilirubin increased | 0 | 3 | 2 | 0 | 5 | 14.7 |
| Back pain | 4 | 1 | 0 | 0 | 5 | 14.7 |
| Productive sputum | 4 | 0 | 0 | 0 | 4 | 11.8 |
| Dry mouth | 3 | 0 | 0 | 0 | 3 | 8.8 |
| Thromboembolic event | 1 | 2 | 0 | 0 | 3 | 8.8 |
| Peripheral sensory neuropathy | 3 | 0 | 0 | 0 | 3 | 8.8 |
| Leg pain | 3 | 0 | 0 | 0 | 3 | 8.8 |
| Productive cough | 3 | 0 | 0 | 0 | 3 | 8.8 |
| Abdominal discomfort | 2 | 0 | 0 | 0 | 2 | 5.9 |
| Dyspepsia | 1 | 1 | 0 | 0 | 2 | 5.9 |
| Peritonitis | 0 | 1 | 1 | 0 | 2 | 5.9 |
| Infections and infestations - Other, specify | 0 | 1 | 1 | 0 | 2 | 5.9 |
| Abdominal distension | 0 | 2 | 0 | 0 | 2 | 5.9 |
| Dizziness | 2 | 0 | 0 | 0 | 2 | 5.9 |
| Flank pain | 2 | 0 | 0 | 0 | 2 | 5.9 |
| Headache | 2 | 0 | 0 | 0 | 2 | 5.9 |
| Cholecystitis | 0 | 1 | 0 | 0 | 1 | 2.9 |
| Epigastric pain | 1 | 0 | 0 | 0 | 1 | 2.9 |
| Ascites | 0 | 1 | 0 | 0 | 1 | 2.9 |
| Hiccup | 1 | 0 | 0 | 0 | 1 | 2.9 |
| Alkaline phosphatase increased | 0 | 0 | 1 | 0 | 1 | 2.9 |
| Chest pain | 1 | 0 | 0 | 0 | 1 | 2.9 |
| Pain (hip) | 0 | 1 | 0 | 0 | 1 | 2.9 |
| Urinary retention | 1 | 0 | 0 | 0 | 1 | 2.9 |
| Pneumonitis | 0 | 0 | 1 | 0 | 1 | 2.9 |
| Upper gastrointestinal hemorrhage | 0 | 0 | 1 | 0 | 1 | 2.9 |
| Presyncope | 1 | 0 | 0 | 0 | 1 | 2.9 |
| Delirium | 0 | 1 | 0 | 0 | 1 | 2.9 |
| Creatinine increased | 1 | 0 | 0 | 0 | 1 | 2.9 |
| Eye pain | 1 | 0 | 0 | 0 | 1 | 2.9 |
| Neck pain | 1 | 0 | 0 | 0 | 1 | 2.9 |
| Insomnia | 1 | 0 | 0 | 0 | 1 | 2.9 |
| Hypokalemia | 0 | 0 | 0 | 1 | 1 | 2.9 |
| Gingival pain | 1 | 0 | 0 | 0 | 1 | 2.9 |
| Shoulder pain | 1 | 0 | 0 | 0 | 1 | 2.9 |
| Herpes zoster | 1 | 0 | 0 | 0 | 1 | 2.9 |
| Altered mentality | 0 | 0 | 1 | 0 | 1 | 2.9 |
| Blood tinged sputum | 1 | 0 | 0 | 0 | 1 | 2.9 |

G, grade.

**Supplementary Table S4. Mutations in the RAS/RAF/MEK/ERK pathway**

|  | Mutation site |
| --- | --- |
| Patient 1 | KRAS(G12D) |
| Patient 2 | MAP2K1(E203K) |
| Patient 3 | KRAS(G12A) |
| Patient 4 | KRAS(G12C) |
| Patient 5 | NRAS(Q61L) |
| Patient 6 | KRAS(G12V) |
| Patient 7 | KRAS (G12V) |
| Patient 8 | KRAS(G12V) |
| Patient 9 | KRAS(G12D) |
| Patient 10 | MAP2K1(E203V) |

**Supplementary Table S5. Tumor response, progression-free survival, and overall survival according to mutation status within the RAS/RAF/MEK/ERK pathway**

|  | **Mutant type (n = 10)** | **Wild type (n = 16)** | **P value** |
| --- | --- | --- | --- |
| **Response** |  |  |  |
| Complete response | 0 (0.0%) | 0 (0.0%) | 0.028 |
| Partial response | 4 (40.0%) | 2 (12.5%) |  |
| Stable disease | 6 (60.0%) | 9 (56.2%) |  |
| Progressive disease | 0 (0.0%) | 5 (31.2%) |  |
| **Objective response rate** | 40.0% | 12.5% |  |
| **Disease control rate** | 100.0% | 68.8% |  |
| **Progression-free survival** | 5.4 months (95% CI, 4.4-NR) | 3.5 months (95% CI, 2.6-5.7) | 0.010 |
| **Overall survival** | 10.8 months (95% CI, 7.4-NR) | 5.9 months (95% CI, 3.8-NR) | 0.160 |

CI, confidence interval; NR, not reached.

**Supplementary Table S6. Quality of life as determined by the EORTC-QLQ- C30**

|  | Baseline score (SD) | Difference from baseline after 1 cycle | Difference from baseline after 2 cycle | Difference from baseline after 4 cycle | Difference from baseline after 6 cycle | Difference from baseline after 8 cycle | Difference from baseline after 11 cycle | Difference from baseline after 14 cycle | Difference from baseline at best score (paired) | 95% Confidence Interval of the Difference | p value (paired t test between baseline and best score) |
| --- | --- | --- | --- | --- | --- | --- | --- | --- | --- | --- | --- |
| Global health status/QoL | 53.2 (20.3) | -5.4 | -1.3 | -4.9 | -5.3 | -6.0 | -7.4 | -7.4 | 5.0 | -1.4, 11.4 | 0.119 |
| Physical functioning | 69.4 (17.6) | 0.1 | 4.3 | -10.4 | -4.6 | -0.5 | 0.6 | -2.7 | 2.3 | -3.9, 8.5 | 0.455 |
| Role functioning | 61.8 (23.4) | 1.4 | -4.2 | -18.5 | -18.1 | -0.7 | -3.5 | 4.9 | 8.0 | 0.9, 15.2 | 0.028 |
| Emotional functioning | 78.3 (18.5) | -3.6 | 0.5 | -8.9 | -16.8 | -0.5 | -45.0 | -36.6 | 1.8 | -7.1, 10.7 | 0.681 |
| Cognitive functioning | 83.3 (20.7) | -2.3 | -3.8 | -11.1 | -12.5 | -11.1 | -25.0 | -8.3 | 0.6 | -7.7, 8.9 | 0.879 |
| Social functioning | 64.6 (28.2) | 0.4 | 5.1 | -6.3 | -8.4 | -3.5 | -14.6 | -14.6 | 9.9 | -2.4, 22.2 | 0.111 |
| Fatigue | 42.8 (23.9) | 0.5 | 3.2 | 8.3 | 7.2 | 5.3 | 7.2 | -9.5 | -4.6 | -12.3, 3.1 | 0.232 |
| Nausea and vomiting | 15.2 (15.2) | 7.6 | 3.0 | 3.7 | -2.7 | -9.6 | -15.2 | 43.1 | 0.0 | -9.4, 9.4 | 1.000 |
| Pain | 33.3 (25.6) | 0.0 | -6.0 | 0.0 | 2.1 | -11.1 | 16.7 | 0.0 | -10.6 | -20.6, -0.5 | 0.039 |
| Dyspnea | 25.5 (26.0) | 8.9 | 0.3 | 7.8 | 7.8 | 18.9 | 7.8 | 41.2 | -2.2 | -13.0, 8.6 | 0.677 |
| Insomnia | 27.5 (29.0) | 4.7 | -0.2 | -3.1 | 14.2 | -5.3 | 5.8 | 39.2 | -4.6 | -18.9, 9.7 | 0.515 |
| Appetite loss | 40.2 (31.5) | 3.1 | 2.2 | 8.7 | 1.5 | -18.0 | -23.5 | -23.5 | -7.8 | -22.6, 7.1 | 0.293 |
| Constipation | 21.6 (30.6) | 9.5 | 5.7 | 11.7 | 7.6 | -21.6 | -4.9 | -21.6 | 1.1 | -9.5, 11.7 | 0.831 |
| Diarrhea | 4.9 (12.0) | 10.7 | 7.2 | 8.4 | 11.8 | 6.2 | -4.9 | -4.9 | 6.7 | -3.4, 16.7 | 0.184 |
| Financial difficulties | 24.5 (27.6) | -6.1 | -4.8 | 8.8 | 4.7 | -13.4 | 25.5 | 42.2 | -8.0 | -15.4, -0.7 | 0.032 |

SD, standard deviation; QOL, Quality of life.

**Supplementary Table S7. Quality of life by EQ5D**

| . | Baseline n (%) | | After 1 cycle | | After 2 cycles | | After 4 cycles | | After 6 cycles | | After 8 cycles | | After 11 cycles | | After 14 cycles | |
| --- | --- | --- | --- | --- | --- | --- | --- | --- | --- | --- | --- | --- | --- | --- | --- | --- |
|  | n | % | n | % | n | % | n | % | n | % | n | % | n | % | n | % |
| Mobility | | | | | | | | | | | | | | | | |
| 1 | 13 | 38.2 | 12 | 40.0 | 6 | 27.3 | 3 | 20.0 | 2 | 25.0 | 0 | 0.0 | 0 | 0.0 | 0 | 0.0 |
| 2 | 10 | 29.4 | 9 | 30.0 | 11 | 50.0 | 4 | 26.7 | 3 | 37.5 | 2 | 66.7 | 0 | 0.0 | 0 | 0.0 |
| 3 | 10 | 29.4 | 7 | 23.3 | 3 | 13.6 | 4 | 26.7 | 2 | 25.0 | 1 | 33.3 | 2 | 100.0 | 2 | 100.0 |
| 4 | 1 | 2.9 | 2 | 6.7 | 2 | 9.1 | 4 | 26.7 | 1 | 12.5 | 0 | 0.0 | 0 | 0.0 | 0 | 0.0 |
| 5 | 0 | 0.0 | 0 | 0.0 | 0 | 0.0 | 0 | 0.0 | 0 | 0.0 | 0 | 0.0 | 0 | 0.0 | 0 | 0.0 |
| Self-care | | | | | | | | | | | | | | | | |
| 1 | 27 | 79.4 | 24 | 80.0 | 14 | 63.6 | 7 | 46.7 | 6 | 75.0 | 1 | 33.3 | 1 | 50.0 | 1 | 50.0 |
| 2 | 7 | 20.6 | 6 | 20.0 | 7 | 31.8 | 5 | 33.3 | 2 | 25.0 | 2 | 66.7 | 1 | 50.0 | 1 | 50.0 |
| 3 | 0 | 0.0 | 0 | 0.0 | 1 | 4.5 | 3 | 20.0 | 0 | 0.0 | 0 | 0.0 | 0 | 0.0 | 0 | 0.0 |
| 4 | 0 | 0.0 | 0 | 0.0 | 0 | 0.0 | 0 | 0.0 | 0 | 0.0 | 0 | 0.0 | 0 | 0.0 | 0 | 0.0 |
| 5 | 0 | 0.0 | 0 | 0.0 | 0 | 0.0 | 0 | 0.0 | 0 | 0.0 | 0 | 0.0 | 0 | 0.0 | 0 | 0.0 |
| Usual activities | | | | | | | | | | | | | | | | |
| 1 | 11 | 32.4 | 8 | 26.7 | 6 | 27.3 | 2 | 13.3 | 1 | 12.5 | 0 | 0.0 | 0 | 0.0 | 0 | 0.0 |
| 2 | 16 | 47.1 | 14 | 46.7 | 11 | 50.0 | 6 | 40.0 | 4 | 50.0 | 3 | 100.0 | 0 | 0.0 | 0 | 0.0 |
| 3 | 5 | 14.7 | 6 | 20.0 | 3 | 13.6 | 4 | 26.7 | 1 | 12.5 | 0 | 0.0 | 2 | 100.0 | 2 | 100.0 |
| 4 | 2 | 5.9 | 2 | 6.7 | 2 | 9.1 | 3 | 20.0 | 1 | 12.5 | 0 | 0.0 | 0 | 0.0 | 0 | 0.0 |
| 5 | 0 | 0.0 | 0 | 0.0 | 0 | 0.0 | 0 | 0.0 | 1 | 12.5 | 0 | 0.0 | 0 | 0.0 | 0 | 0.0 |
| Pain/Discomfort | | | | | | | | | | | | | | | | |
| 1 | 8 | 23.5 | 4 | 13.3 | 8 | 36.4 | 2 | 13.3 | 2 | 25.0 | 1 | 33.3 | 0 | 0.0 | 0 | 0.0 |
| 2 | 15 | 44.1 | 17 | 56.7 | 9 | 40.9 | 8 | 53.3 | 3 | 37.5 | 2 | 66.7 | 1 | 50.0 | 2 | 100.0 |
| 3 | 9 | 26.5 | 6 | 20.0 | 3 | 13.6 | 4 | 26.7 | 2 | 25.0 | 0 | 0.0 | 1 | 50.0 | 0 | 0.0 |
| 4 | 2 | 5.9 | 3 | 10.0 | 1 | 4.5 | 1 | 6.7 | 1 | 12.5 | 0 | 0.0 | 0 | 0.0 | 0 | 0.0 |
| 5 | 0 | 0.0 | 0 | 0.0 | 1 | 4.5 | 0 | 0.0 | 0 | 0.0 | 0 | 0.0 | 0 | 0.0 | 0 | 0.0 |
| Anxiety/Depression | | | | | | | | | | | | | | | | |
| 1 | 17 | 50.0 | 15 | 50.0 | 14 | 63.6 | 5 | 33.3 | 3 | 37.5 | 1 | 33.3 | 0 | 0.0 | 0 | 0.0 |
| 2 | 15 | 44.1 | 7 | 23.3 | 3 | 13.6 | 7 | 46.7 | 4 | 50.0 | 2 | 66.7 | 2 | 100.0 | 2 | 100.0 |
| 3 | 1 | 2.9 | 7 | 23.3 | 4 | 18.2 | 3 | 20.0 | 1 | 12.5 | 0 | 0.0 | 0 | 0.0 | 0 | 0.0 |
| 4 | 1 | 2.9 | 1 | 3.3 | 1 | 4.5 | 0 | 0.0 | 0 | 0.0 | 0 | 0.0 | 0 | 0.0 | 0 | 0.0 |
| 5 | 0 | 0.0 | 0 | 0.0 | 0 | 0.0 | 0 | 0.0 | 0 | 0.0 | 0 | 0.0 | 0 | 0.0 | 0 | 0.0 |
| EQ VAS | | | | | | | | | | | | | | | | |
| Mean | 66.0 | | 62.5 | | 63.9 | | 58.3 | | 64.4 | | 60.0 | | 50.0 | | 47.5 | |
| SD | 20.1 | | 17.1 | | 21.8 | | 17.4 | | 22.3 | | 17.3 | | 0.0 | | 3.5 | |

SD, Standard deviation; VAS, visual analogue scale.
